# END-USER PERSPECTIVES ON POLICIES FOR SOCIAL ROBOTICS WITH AGING APPLICATIONS

**DOI:** 10.1093/geroni/igae098.1113

**Published:** 2024-12-31

**Authors:** Julie Robillard, Jill Dosso, Haiger Ye, Gabriella Guerra, Anna Riminchan

**Affiliations:** University of British Columbia, Vancouver, British Columbia, Canada; University of British Columbia, Vancouver, British Columbia, Canada; University of British Columbia, Vancouver, British Columbia, Canada; University of British Columbia, Vancouver, British Columbia, Canada; University of British Columbia, Vancouver, British Columbia, Canada

## Abstract

Social robots, including those with artificial intelligence (AI)-enabled functionalities, are promising in their potential to support brain health in older adults in both home and healthcare environments, but their benefits must be weighed against ethical considerations and risks. To characterize and evaluate existing guidance in this field, we conducted a content analysis of n = 47 international policies on social robotics and conducted n = 18 semi-structured interviews with lived experience experts within the dementia and care partnership community and professional experts in the field of aging and dementia. Participants viewed social robots as potentially helpful assistive technologies and agreed with existing recommendations around the needs for respect for human rights and dignity, clear and consistent regulation, and public engagement. Participants recommended that policies prioritize cost and accessibility considerations, represent the needs of minority groups, and that they center around issues specific to aging and dementia. They reported that the responsibility for social robot policy development should lie with governments and the healthcare sector and called for increased consultation with end-users and medical professionals. Results also highlighted misalignments between the priorities of the aging and dementia community members and the existing social robot policy landscape. Potential harms and challenges to adoption of social robots were discussed, including discrimination, bias, and deception. Taken together, findings from this two-phase study can inform evidence-based policy recommendation for the development and implementation of AI-enabled social robots for aging.

